# Rapid Metagenomic Detection of *Brucella abortus* During a Two-Case Bovine Abortion Investigation in Inner Mongolia, China

**DOI:** 10.3390/vetsci13060541

**Published:** 2026-05-30

**Authors:** Tianqi Xue, Boyuan Zhang, Ziyan Wang, Yue Ma, Qingchun Shen, Jiabo Ding, Xiaowen Yang

**Affiliations:** 1 Key Laboratory of Jiangsu Preventive Veterinary Medicine, Key Laboratory of Avian Preventive Medicine, Ministry of Education, College of Veterinary Medicine, Yangzhou University, Yangzhou 225009, China; 15052505301@163.com; 2 Key Laboratory of Animal Biosafe Risk Prevention and Control (North), Ministry of Agriculture and Rural Affairs, Institute of Animal Science, Chinese Academy of Agricultural Sciences, Beijing 100193, China; zhangboyuan@caas.cn (B.Z.); princeyan2002@163.com (Z.W.); 18801291321@163.com (Y.M.)

**Keywords:** abortion, brucellosis, *Brucella abortus*, metagenomic sequencing

## Abstract

Abortion in cattle entails substantial economic loss, and rapid identification of abortigenic pathogens is critical for timely on-farm response and reduction in human exposure risk. In 2024, two Holstein cows from a small farm in Inner Mongolia aborted in close succession without an obvious cause. Vulvar swabs from both cows, one afterbirth sample, and whole blood from one aborted fetus were collected. Shotgun metagenomic sequencing was performed, followed by host-read removal, taxonomic profiling with Kraken2, de novo assembly of Brucella-aligned reads, and whole-genome comparison. Serological tests, Gram-stained smears, and Brucella genus- and species-specific qPCR assays were used as orthogonal verification. Putative resistance and virulence determinants were screened against CARD and VFDB. *Brucella* reads were detected in all samples, with the highest relative abundance in the 138-afterbirth (96%). qPCR assays detected *Brucella* DNA and *B. abortus*-specific signals in all four samples. A draft *Brucella* genome was assembled from the 138-afterbirth sample and was phylogenetically placed within *B. abortus*, showing relatedness to previously circulating Chinese lineages. Cows 138 and 198 were RBT-positive with SAT titres of 1:100 (++). No acquired *Brucella* resistance genes were identified in CARD. Within 72 h of sample receipt, *B. abortus* was reported to the farm and local authorities and emergency biosecurity measures were implemented. This field investigation shows that metagenomic sequencing, when combined with conventional serology, microscopy, and targeted qPCR, can support rapid etiological investigation when culture is delayed, hazardous, or biosafety level 3 facilities are unavailable.

## Simple Summary

In this study, we demonstrate the successful application of shotgun metagenomic sequencing for the rapid diagnosis and management of two bovine abortion cases associated with *Brucella abortus* infection in Inner Mongolia, China. Traditional diagnostic methods for brucellosis are time-consuming, often taking a week or longer. Our approach enabled us to detect *B. abortus* DNA and provide preliminary genomic context within 72 h, allowing for prompt intervention measures that effectively prevented the spread of brucellosis to farm personnel.

## 1. Introduction

Cattle abortions pose significant economic challenges because of the long gestation period of bovines and the direct losses associated with fetal death, reduced productivity, and herd-level control measures. Infectious agents are among the most important pathological causes of bovine abortion and include bacteria such as *Brucella* spp. [1], *Salmonella* spp. [2], and *Listeria* spp. [3]), viruses such as bovine viral diarrhea virus (BVDV) [4], and parasites such as *Neospora caninum* [5]). Among these pathogens, *Brucella* spp. is of particular concern because it causes reproductive failure in cattle and poses an occupational zoonotic risk to farmers, veterinarians, and laboratory personnel [6]. Rapid etiological clarification is therefore important not only for herd management but also for human exposure assessment.

Traditional diagnostic methods, including culture, serology, targeted PCR, are informative but may be time-consuming or constrained by sample quality, biosafety requirements, and the need to select specific target pathogens in advance [7]. *Brucella* culture requires appropriate containment and is not always feasible in routine or field-associated investigations. Metagenomic sequencing provides a culture-independent and hypothesis-free approach by analyzing total nucleic acids in clinical or environmental specimens [8]. This approach can identify unexpected or mixed pathogens and can generate ancillary genomic information for source tracing and resistance-gene screening [9,10]. However, metagenomic sequencing should not be considered a replacement for targeted real-time PCR or culture when a known pathogen is strongly suspected and appropriate laboratory capacity is available. Its practical niche is in time-critical field investigations, unknown-cause outbreaks, mixed infections, or situations in which culture is delayed, hazardous, or unavailable. In this study, we evaluated whether shotgun metagenomic sequencing could support rapid identification of the abortigenic agent in a small bovine abortion event under field constraints and integrated the results with serology, microscopy, and targeted qPCR.

## 2. Materials and Methods

### 2.1. Case Presentation

In 2024, two pregnant Holstein cows on a small dairy farm (50 dairy cows) in Inner Mongolia experienced successive abortions with no apparent cause. There were no toxins detected on this farm, no new cattle had been introduced, and no history of exposure to common abortifacient diseases. The two aborted cows, designated 138 and 198, were both primiparous; cow 138 aborted during early pregnancy and cow 198 aborted during mid-pregnancy. The farm had no known history of brucellosis, no recent introduction of new cattle, and no vaccination against brucellosis or other common abortifacient pathogens. BVDV testing performed before this investigation was negative. Because the farm owner suspected an unknown or mixed infectious cause and culture was not promptly feasible under field and biosafety constraints, shotgun metagenomic sequencing was used as a broad, culture-independent diagnostic approach.

### 2.2. Sample Collection

Vulvar swabs were collected from the aborted cows, whole blood from the aborted fetuses (designated blood), and the placenta from one of the cows (designated 138-afterbirth). The specimens were collected by farm personnel and transported to the laboratory for testing; the approximate interval from abortion to laboratory receipt was about 3 days. One milliliter of whole blood was centrifuged at 4000 rpm at room temperature to collect serum. All samples and related extracts were stored at −80 °C.

### 2.3. Shotgun Metagenomic Sequencing and Analysis

DNA was extracted as in a previous study [11]. DNA was randomly sheared to construct paired-end libraries with an average read length of 150 bp using the MGISEQ-2000RS DNA Library Kit (PE150, MGI, China). Sequencing was conducted on the MGISEQ-2000RS platform (BGI, Shenzhen, China). Quality control of the raw data was performed using FastQC (http://www.bioinformatics.babraham.ac.uk/projects/fastqc/, accessed on 3 April 2026) and Trimmomatic (http://www.usadellab.org/cms/?page=trimmomatic, accessed on 3 April 2026) as in the previous study [11]. Reads aligning to the host genome (NCBI RefSeq assembly number GCF_002263795.2) were removed using Bowtie2 [12] and kneaddata (v0.12.2). The remaining reads were analyzed using Kraken2 (v2.17.1) [13] by comparing them to archaea, bacteria, plasmid, viral, fungi, protozoa and UniVec databases to obtain taxonomic abundance profiles. In addition, environmental samples from the farm and swab and blood samples from clinically healthy cattle were collected as negative controls for *Brucella*-specific qPCR; these control samples were negative for *Brucella* DNA. These control samples were not subjected to metagenomic sequencing.

### 2.4. Genome Assembly and Phylogenetic Tree Construction

A reference library was constructed from complete genome sequences of representative *Brucella* species and biovars retrieved from NCBI RefSeq (Table S1). Clean non-host reads were aligned to this library, and Brucella-aligned reads were extracted for de novo assembly using SOAPdenovo2 (v2.0) [14] to obtain contigs. Reads were then mapped back to the contigs, and assembly was optimized based on paired-end and overlap relationships. The draft genome was screened against CARD (https://card.mcmaster.ca, accessed on 3 April 2026) and VFDB (https://www.mgc.ac.cn/VFs, accessed on 3 April 2026) to identify known acquired antimicrobial resistance determinants and virulence-associated genes. Because no bacterial isolate was recovered, genomic screening was interpreted only as an indication of whether known acquired resistance genes were detected and not as phenotypic susceptibility testing.

Genome sequences of Chinese isolates and reference genome of various *Brucella* species and biovars (Table S2) were downloaded for comparative analysis. Whole-genome alignment was performed using MAUVE [15], and phylogenetic trees were constructed to determine species placement and evaluate relatedness to previously reported strains [16]. The phylogenetic analysis was interpreted cautiously because the genome was reconstructed from metagenomic reads rather than from a cultured isolate.

### 2.5. Sample Detection

qPCR assays were performed using Premix Ex Taq (Probe qPCR) (TaKaRa, Japan) with TaqMan probes and *Brucella* genus- [11] and species-specific [16] primers described previously. Samples with clear amplification curves and Ct values below 38 were considered positive (Tianlong, China). VIC and FAM channels were used to record the *Brucella* genus-specific and *B. abortus*-specific signals, respectively.

Serological testing. To verify the analysis results of shotgun metagenomic sequencing results, serological testing was performed for the two aborted cows. Rose Bengal plate agglutination test (RBT) and serum agglutination test (SAT) were performed as in previous studies [17,18]. Because the farm had no history of brucellosis vaccination, vaccine-induced seropositivity was considered unlikely. SAT results were interpreted using the established diagnostic cut-off for bovine brucellosis, with titers of 1:100 or higher and an agglutination reaction of ++ or stronger considered positive.

Smear testing. Fifty microliters of each collected samples were placed on a glass slide, fixed, and Gram-stained using a kit (Solarbio, China). Samples were observed under a microscope (Nikon, Japan).

### 2.6. Statistical Analysis

**Figure 1:**
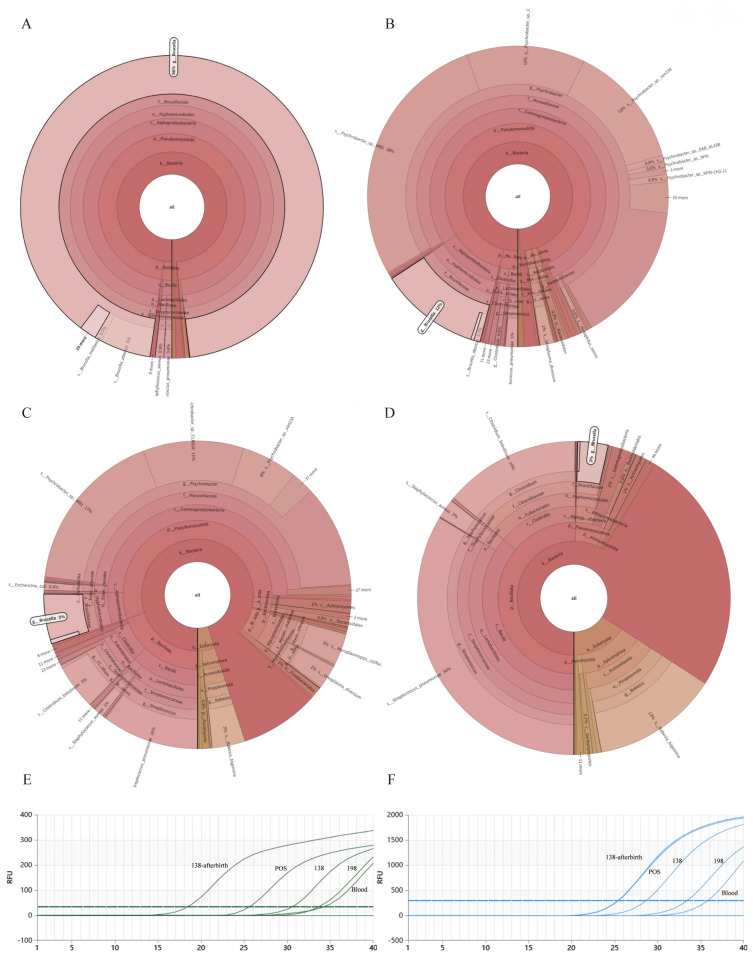
Shotgun metagenomic sequencing and qPCR detection. (**A**–**D**) Taxonomic composition of microorganisms detected in the submitted specimens by Kraken2. Different colors indicate different bacterial genera. Abundance refers to the relative percentage of each detected taxon among classified microbial reads (i.e., reads assigned to bacteria, archaea, viruses, or fungi, after removal of host reads). Only genera with relative abundance ≥0.5% are labeled; lower-abundance genera are pooled into “Others”. (**A**) 138-afterbirth sample; (**B**) 138 sample; (**C**) 198 sample; (**D**) Blood sample. (**E**,**F**) qPCR detection results. Samples with clear exponential amplification curves and cycle threshold (Ct) values <38 were considered positive. VIC channel recorded genus-specific signals (**E**); FAM channel recorded B. abortus-specific signals (**F**). Negative controls (extraction blank and no-template control) showed no amplification in either channel.

**Figure 2:**
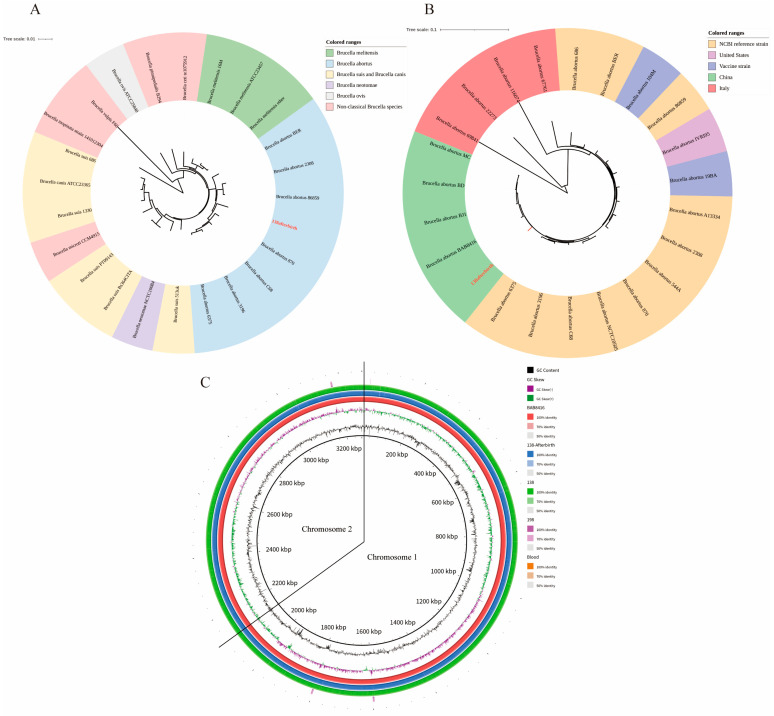
Phylogenetic trees of the strain. (**A**) Comparison with different Brucella species and biovars. (**B**) Comparison with various *B. abortus* strains. (**C**) Alignment of sample genome sequences with *B. abortus* BAB8416.

Analyses were descriptive because this was a two-case field investigation rather than a herd-level epidemiological study. Taxonomic abundance plots (Figure 1A–D) were generated from Kraken2 outputs, the qPCR amplification curves were generated by the instrument (Figure 1E,F), genome comparisons and phylogenetic visualizations were generated using MAUVE and iTOL (https://itol.embl.de, accessed on 3 April 2026) (Figure 2A,B), and summary figures were edited in R (Figure 2C). No inferential statistical testing was performed.

## 3. Results

### 3.1. Detection of Brucella DNA in Abortion-Associated Specimens

To rapidly diagnose the cause of abortion, microbiota in samples by metagenomic sequencing was analyzed. Each sample yielded over 40 Gb of raw data, with approximately 400 Mb~4 Gb of clean data remaining after host alignment removal. Kraken2 analysis revealed that the the 138-afterbirth sample was predominantly composed of Brucellaceae (Figure 1A). *Brucella*-mapped sequences were detected in all remaining samples (Figure 1B–D). The blood sample primarily contained environmental microorganisms, with Brucella spp. at 3% abundance (Table 1).

To verify the results of metagenomic sequencing analysis, we performed serological testing, Gram-stained smear microscopy, and targeted qPCR. Both aborted cows were positive by RBT, and the SAT antibody titers were 1:100, ++. The bacteria were approximately 0.6–1.8 µm, spherical to rod-shaped, arranged singly, and lacked capsules and spores, which was consistent with *Brucella* spp. qPCR assays targeting *Brucella* genus-specific and *B. abortus*-specific markers yielded amplification curves in all four submitted specimens.

### 3.2. Draft Genome Recovery and Phylogenetic Placement as B. abortus

To trace the *B. abortus* strain, this study analyzed the metagenomic data, assembled and constructed the phylogenetic tree. De novo assembly of the 138-afterbirth sample resulted in a genome of approximately 3.2 Mb consisting of 236 contigs. N50 is 138,170, and the max contig is 283,871 bp. GC content is 57.2%. Phylogenetic analysis indicated that the assembled genome was assigned to *B. abortus* (Figure 2A). Further comparison with previously reported *B. abortus* genomes showed clustering with Chinese isolates (Figure 2B), suggesting relatedness to previously circulating local lineages rather than demonstrating dominance of this lineage.

Contigs from the 198 and blood samples did not cover the entire *B. abortus* genome, and the assembly from the 138 sample included sequences from other bacteria due to their relative abundances (Figure 2C).

### 3.3. Operational Response and Short-Term Human Follow-Up

No known acquired *Brucella* resistance determinants cataloged in CARD were identified in the draft assembly, and no exogenous virulence gene modules were detected in the VFDB screen. These genomic findings do not substitute for phenotypic susceptibility testing. In particular, absence of a known acquired resistance gene does not exclude resistance mediated by novel mutations or mechanisms not represented in the database.

Within 72 h of sample receipt, the detection of *B. abortus* was reported to the farm and local authorities. Three potentially exposed farm workers (two workers had contact with abortion materials while wearing gloves and masks) were advised to receive post-exposure prophylaxis according to local medical practice and published laboratory-exposure recommendations [19]. The regimen consisted of oral doxycycline plus rifampicin for 3 weeks: doxycycline 100 mg twice daily and rifampicin 600 mg once daily. Enhanced disinfection and biosecurity measures were also implemented on the farm. Human brucellosis testing and follow-up were performed by the local Center for Disease Control and Prevention rather than by our laboratory. Four weeks later, farm workers reported by telephone that all three workers had negative Brucella serology and no brucellosis-compatible symptoms during the follow-up period.

## 4. Discussion

This field investigation demonstrates that shotgun metagenomic sequencing can support rapid etiological assessment of bovine abortion when the causative agent is uncertain and culture is not promptly feasible. Traditional methods for diagnosing brucellosis can take a week or longer, but metagenomic sequencing allowed researchers to identify and trace the pathogen within three days, enabling timely intervention. The high relative abundance of *Brucella* reads suggested a substantial bacterial DNA burden in the 138-afterbirth sample, aligning with previous findings [20]. The blood sample contained relatively large concentrations of *Bacillus cereus*. Because these bacteria are common environmental organisms and the specimen was transported from the farm after abortion, their presence may reflect post-abortion contamination or overgrowth. To address the possibility that low-abundance *Brucella* reads represented environmental contamination, we performed additional control assays: environmental samples from the farm and swab and blood samples from clinically healthy cattle were negative by *Brucella* qPCR, whereas all submitted abortion-associated specimens were positive by genus- and species-specific qPCR. In addition, culture and 16S sequencing of colonies from a healthy cow vaginal swab mainly identified *Bacillus cereus*, supporting the interpretation that *Bacillus* detection in some samples reflected environmental or commensal contamination rather than the primary abortigenic agent.

In this study, a draft genome was assembled from clean data after removing reads aligned to the host genome. The genome was reconstructed from metagenomic reads and not from a cultured isolate. The 138-afterbirth sample had a high abundance of *Brucella* spp., allowing us to obtain the draft genome of the causative agent. The assembled data of the other samples could only be aligned to partial sequences in the *Brucella* genome due to lower abundance and the presence of other microorganisms. This limitation illustrates a key challenge of metagenomic assembly from non-sterile field specimens. Metagenomic sequencing detects all microorganisms, and assembly is difficult when there are many species present. The assembled draft genome from the 138-afterbirth sample facilitated phylogenetic analysis, revealing close relations to Chinese *B. abortus* isolates, such as BAB8416 [21], which was isolated from the blood of Baotou brucellosis patients in Inner Mongolia in 2013. Other closely related isolates include *B. abortus* BD (BioSample number SAMN07508059) and *B. abortus* MC (BioSample number SAMN07508100), which were isolated from abortion secretions of cows; and *B. abortus* BJ1 (BioSample number SAMN10238131), which was isolated from elk. These findings suggest relatedness to previously circulating local lineages. However, because the genome was reconstructed from metagenomic reads and no isolate was obtained, the phylogenetic result should be interpreted as contextual evidence rather than definitive source-tracing or evidence that the lineage is dominant in China.

In the present study, CARD screening did not identify known acquired resistance genes in the draft assembly. This result should be interpreted cautiously: it does not establish phenotypic susceptibility and cannot be used to infer the effectiveness of human prophylaxis. Definitive susceptibility profiling would require recovery of a viable isolate and standardized antimicrobial susceptibility testing.

The present study also highlights the appropriate scope of metagenomic sequencing in veterinary diagnostics. For routine brucellosis diagnosis in laboratories with established capacity, targeted real-time PCR and serological testing remain faster, cheaper, and easier to interpret. Metagenomic sequencing is most valuable when the etiology is unknown, when mixed infection is suspected, when conventional assays have failed, or when culture is delayed or unsafe because biosafety level 3 facilities are unavailable. The main limitations of this investigation are the small sample size, absence of isolate recovery, limited availability of fetal tissues, lack of metagenomic sequencing for negative controls, lack of phenotypic antimicrobial susceptibility testing and short-term human follow-up. These limitations have been acknowledged, and the causal and operational conclusions have been restricted accordingly. Another limitation of this study is that approximately three days elapsed between sample collection and laboratory testing, which increased the risk of contamination, degradation and post-abortion microbial overgrowth. Future multicentre studies with larger sample sizes are needed to validate the practical value of metagenomic sequencing for the diagnosis of bovine abortion.

## 5. Conclusions

In this two-case bovine abortion field investigation, shotgun metagenomic sequencing rapidly identified *B. abortus* DNA and provided genomic context suggesting relatedness to previously circulating local lineages. The metagenomic findings were supported by qPCR, serology, and smear microscopy, but were not validated by isolate recovery. When integrated with routine diagnostic methods, metagenomics can serve as a useful adjunct for time-critical veterinary investigations, particularly when the causative agent is unknown or when culture is constrained by biosafety or logistics.

## Figures and Tables

**Table 1:** Number of aligned reads of shotgun metagenomic sequencing.

Sample	Number of Total Reads	Number of Aligned Reads	Relative Abundance	Genus
138-afterbirth	2,519,577	2,412,475	95.7%	Brucella
		22,679	0.9%	Staphylococcus
		12,988	0.5%	Clostridium
138	9,725,532	7,323,301	75.3%	Psychrobacter
		1,139,276	12.0%	Brucella
		395,505	4.1%	Clostridium
		158,271	1.6%	Ureaplasma
198	815,527	454,909	55.8%	Psychrobacter
		161,890	19.9%	Clostridium
		83,986	10.3%	Streptococcus
		42,456	5.2%	Brucella
Blood	380,229	127,783	33.6%	Streptococcus
		53,610	14.1%	Clostridium
		49,460	13.0%	Babesia
		11,756	3.1%	Brucella

## Data Availability

The data that support the findings of this study are openly available in SRA database at https://www.ncbi.nlm.nih.gov/bioproject/PRJNA1468533/, accessed on 3 April 2026.

## Supplementary Materials

The following supporting information can be downloaded at https://www.mdpi.com/article/10.3390/vetsci13060541/s1, Table S1: Different species of *Brucella* genome used in this study; Table S2: Different biovar of *B. abortus* genome used in this study.
